# Auditory Discrimination—A Missing Piece of Speech and Language Development: A Study on 6–9-Year-Old Children with Auditory Processing Disorder

**DOI:** 10.3390/brainsci13040606

**Published:** 2023-04-03

**Authors:** Anna Guzek, Katarzyna Iwanicka-Pronicka

**Affiliations:** 1 The Outpatient of Speech Therapy, The Children’s Memorial Health Institute, 04-736 Warsaw, Poland; 2 Department of Medical Genetics, The Children’s Memorial Health Institute, 04-736 Warsaw, Poland

**Keywords:** auditory processing disorder, APD, CAPD, phoneme discrimination, categorical perception, auditory perception, frequency pattern discrimination, FPT, speech development, language development

## Abstract

Auditory discrimination, the hearing ability crucial for speech and language development, allowing one to perceive changes in volume, duration and frequency of sounds, was assessed for 366 participants with normal peripheral hearing: 220 participants with auditory processing disorders (APD) and 146 typically developing (TD) children, all aged 6–9 years. Discrimination of speech was tested with nonsense words using the phoneme discrimination test (PDT), while pure tones—with the frequency pattern test (FPT). The obtained results were statistically analyzed and correlated. The median of the FPT results obtained by participants with APD was more than twice lower than those of TD (20% vs. 50%; *p* < 0.05), similarly in the PDT (21 vs. 24; *p* < 0.05). The FPT results of 9-year-old APD participants were worse than the results of TD 6-year-olds (30% vs. 40%; *p* < 0.05), indicating that the significant FPT deficit strongly suggests APD. The process of auditory discrimination development does not complete with the acquisition of phonemes but continues during school age. Physiological phonemes discrimination is not yet equalized among 9-year-olds. Nonsense word tests allow for reliable testing of phoneme discrimination. APD children require testing with PDT and FPT because both test results allow for developing individual therapeutic programs.

## 1. Introduction

The complex process of speech and language development is biologically and socially conditioned. From the biological point of view, proper language acquisition requires correct sound reception. This—in turn—is determined by the correct operation of the auditory system, starting from the peripheral part, encompassing both conductive and perceptive hearing apparatus.

Auditory information is processed and relayed by the upper part of the auditory pathway directly to the cortex. Complex auditory functions, such as sound localization and lateralization, auditory discrimination, sound pattern recognition, temporal auditory processing, dichotic listening and the ability to understand distorted speech or speech in the presence of background noise [1], develop along with the maturation of the nervous system. Auditory processing disorders (APD) are conditions in which the brain, while obtaining correct auditory information, is unable to process sounds correctly, thus causing difficulties in understanding and memorizing sounds, including verbal information and—therefore—the language input. Thus, imbalanced development of the auditory pathway in childhood can constitute a significant obstacle to proper language development and—furthermore—could cause subsequent difficulties in reading and writing [2,3,4,5]. Difficulties in processing auditory stimuli can occur in both the ascending and descending auditory pathways and may be related to impaired neuronal synchronization, atypical functional asymmetry of the hemispheres, or limited interhemispheric transmission [6]. The diagnosis of APD is based on the presence of significant deficits in auditory functions, as seen in the results of a minimum of two evaluation tests (verbal and non-verbal stimuli), with simultaneous undisturbed cognitive level and normal hearing status [1]. APD is most commonly diagnosed in early school-age children with normal peripheral hearing who are struggling with auditory problems.

The ability to discriminate sounds, as one of the higher auditory functions, is attributed a special role in the development of speech. This function encompasses the perception of differences in changing volume and duration of sounds, as well as their frequency—which constitutes the most distinctive feature of sounds [7,8,9,10]. For the assessment of the level of this auditory function, electrophysiological methods are used, such as Mismatch Negativity (MMN), as well as behavioral methods, for example, a Frequency Pattern Test (FPT), both with the use of non-verbal stimuli [11]. Difficulties in sound frequency differentiation can lead to discrimination deficits of speech sounds—phonemes, and consequently problems in distinguishing similar-sounding words—therefore, understanding speech as such.

The intensive development of phoneme discrimination abilities normally begins at the end of the first year of life and is socially conditioned by stimulation by the mother tongue [4,9,12,13]. Apart from difficulties in understanding speech, the deficits in phoneme discrimination skills may cause delay or impaired language development, articulation disorders and problems with reading or writing [2,14,15,16]. The results of research on the ability to discriminate phonemes in various language systems performed over the world do not precisely answer the question of the end of the development of the function. An overview of the available literature on the subject is presented in the Discussion section. Difficulties with discriminating between phonemes are among symptoms of APD [1,17], and the need to assess this skill is accentuated by the APD diagnosing guidelines of audiological associations [1,18] and by the authors of several studies [4,19,20].

The aim of our study is to examine the ability to discriminate sounds by children with and without APD, as assessed through the FPT and phoneme discrimination test (PDT) administered to participants of our study. Additionally, we are trying to determine the need to modify the APD diagnostic standards by including the PDT test in the battery of regularly administrated APD diagnostic tests.

We hypothesized that children with APD would perform poorly on phoneme discrimination tasks. We also hypothesized that the development of phoneme discrimination skills in typically developing children does not end with the acquisition of phonemes but continues even at school age.

## 2. Materials and Methods

The study included a group of 366 children—speakers of the Polish language, aged 6–9 years old, all with normal results of basic hearing tests. This group included both participants diagnosed with APD and typically developing children. The exclusion criteria comprised intellectual disability (based on information obtained from medical records), developmental disorders (such as autism spectrum disorders), neurological problems (in this epilepsy), and congenital disorders with an established genetic basis. Our research group did not include any SLI-diagnosed participants. The participants of the study were recruited from patients of the Audiology Department of the Children’s Memorial Health Institute (CMHI) in Warsaw and students of 3 Warsaw primary schools. Patients recruited from the Audiology Department were referred for hearing testing due to auditory difficulties or the suspicion of APD.

For the study group, higher auditory functions were assessed through behavioral psychoacoustic tests. The obtained results were anonymized and evaluated in accordance with the diagnostic criteria of the American Speech-Language-Hearing Association [1]. Eventually, the subjects were divided into 2 groups:

- The APD group: included 220 participants with APD (with more than 1 result of the psychoacoustic tests below the norm);
- The TD (typically developing) group: comprised 146 typically developing participants, not diagnosed with APD (each child obtained a maximum of 1 result below the norm in the psychoacoustic tests). This was considered a control group.

Table 1 shows the number of participants and percentage distribution of sexes, in individual age groups, for both APD and TD groups.

The distribution of sexes in both study groups was similar: boys constituted 2/3 of each group. Since the development of auditory functions does not differ between sexes, the collected data was not analyzed by gender [21,22].

All participants were examined using the same protocol, and the tests were performed within 1 day at the Department of Audiology of the CMHI.

1. Hearing status was assessed with pure-tone audiometry (PTA), speech audiometry (AC40, Interacoustics, Denmark, with TDH39 headphones), tympanometry and otoacoustic emissions (Titan, Interacoustics, Denmark). Normal hearing sensitivity was diagnosed when the hearing thresholds in PTA were below 20 dB for the tested frequency range (125–8000 Hz). The result of the type A tympanometry curve was understood as confirmation of the correct function of the middle ear, according to Jerger’s [23] criteria. The regular inner ear condition was determined with the registration of the otoacoustic emissions signal from both ears. Clinical Distortion Product Otoacoustic Emissions (DPOAEs) were used for the diagnostics;
2. Higher auditory functions were assessed with a battery of psychoacoustic tests available online on the ATS Neuroflow platform. The battery of tests includes:
  - Adaptive Speech in Noise Test (ASPN), a test assessing understanding of speech in noise (the presented study used multitalker babble);
  - Dichotic Digits Test with distracted attention (DDT), which assesses the maturity of the auditory system in its central part and enables identification of the cerebral hemisphere dominance in terms of verbal stimuli;
  - The Frequency Pattern Test (FPT), which assesses the ability to discriminate sound frequencies, the level of auditory short-term memory, and the level of functioning of the right cerebral hemisphere [24];

1. The testing procedure was always conducted by the same certified Neuroflow provider specialist in an acoustically treated room, using an AC40 audiometer (Interacoustics, Denmark) and TDH39 headphones (Interacoustics, Denmark). Auditory stimuli were presented for both ears at a comfortable volume level of 60 dB for both ears. One complete examination procedure lasted about 20 min. The tests were carried out in the same order for each subject. Each participant’s caregiver completed a symptom questionnaire (constituting part of the battery used in the study). The study was conducted according to the procedure already described [24] (Appendix A);
2. The assessment of phoneme discrimination was carried out using a standardized test included in the battery of phonological tests of the Educational Research Institute [25]. The test consists of 25 pairs of nonsense words with the same syllable structure. Among these words, 18 pairs differ with 1 distinctive feature (1 phoneme), and in the remaining 7 pairs, the nonsense words are the same (constituting control samples, according to the authors of the test). Phonological oppositions concern voicing (5 pairs), manner of articulation (4 pairs), place of articulation (7 pairs) and nasality (2 pairs of vowels), which are a contrastive feature of the Polish language. Nonsense words used in this study allowed us to eliminate the influence of the semantic factor. The full list of the pairs of words used, together with their phonetic notation, is presented in Table A1 in Appendix B. One speech-language pathologist—a female—conducted the phoneme discrimination test for each child: pairs of words were pronounced in the same order, with the natural speaking rate and intonation. Having heard the words, participants were required to assess whether they were the same or different, scoring 1 point for each correctly identified pair. The maximum test score was then 25 points. The test’s duration was approximately 5 min.

Statistical analysis was performed using MS Excel and Statistica for Windows. Differences were considered statistically significant for *p* < 0.05. The Kolmogorov-Smirnov and Lilliefors tests (Appendix C Table A2) were used to evaluate the normality of distributions. Since some of the analyzed data did not have a normal distribution, the non-parametric Kruskal–Wallis ANOVA test and the post hoc multiple comparison tests were used to analyze the differences between the groups. The correlations between the variables were assessed based on the results of Spearman’s rank test and Pearson’s test (a parametric test was also used due to the large size of study groups and the need to obtain precise results) [26]. The correlation coefficient *r* was considered statistically significant for *p* < 0.05. To determine the strength of the relationship between the examined variables, the scale used in the statistical analysis was adopted (Table 2 below) [27].

## 3. Results

We compared the results of FPT obtained by the participants of both study groups. Basic statistics: mean, SD, median, minimum and maximum are presented in Table 3 below.

The results of FPT were also analyzed in reference to the age norms for 6, 7, 8, and 9-year-olds and are presented in Table 4 below as a percentage of the age standard. Reference values for FPT results are provided in Table A3 in Appendix D. Such an approach to FPT results allows for comparing the depth of deficits between age groups. The median of the results of APD participants was more than twice lower than the median for the TD participants. Similar disproportions were observed in all age groups.

The results did not differ between age groups within each tested group, as demonstrated by the non-parametric Kruskal–Wallis test for *p* = 0.5316 (for the APD group) and *p* = 0.3937 (for the TD group). The results differed between the APD and TD study groups, as shown by Kruskal–Wallis analysis at *p* = 0.0000.

**Figure 1:**
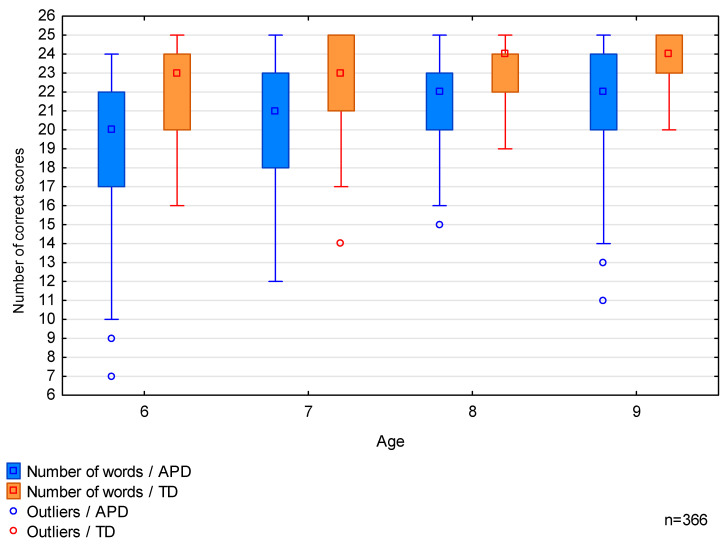
Comparison of the Phoneme Discrimination Test (PDT) results, among age groups, in auditory processing disorder (APD) and typically developing (TD) groups.

The results of the phoneme discrimination test for both study groups are presented in Table 5 and Figure 1 below.

The results of the PDT for TD participants exceeded those of APD participants. The difference was statistically significant, as confirmed by the Kruskal-Wallis test for *p* = 0.0000.

Subsequently, the Kruskal-Wallis test showed that the PDT results differed between age groups within each study group, as presented in Table 6 below.

**Figure 2:**
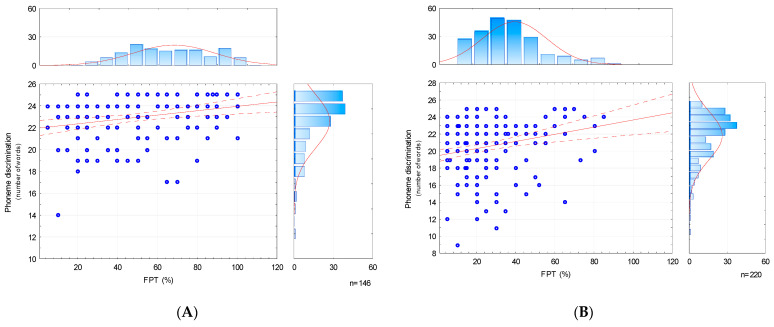
Correlation between Frequency Discrimination Test (FPT) and PDT results, (**A**) TD and (**B**) APD groups.

Next, the correlations between the results obtained in the FPT and PDT were assessed. For this, Spearman’s rank correlation and Pearson’s correlation tests were performed. The correlation coefficient *r* in both tests was on a similar level. Detailed test results are presented in Table 7 and Figure 2, below.

The results obtained in both groups were statistically significant. The correlation coefficient was at least at the *fair* level, and it was slightly higher in the TD group.

## 4. Discussion

The relationship between higher auditory functions and language development has been the subject of research for years [3,4,28,29]. The ability to discriminate phonemes, one of the elements of auditory perception, determines correct understanding, acquisition of phonemes and speaking [30]. This research was inspired by a long-time clinical observation of children, which indicated that the categorical perception of phonemes might remain immature even at school age. The observation has contradicted some literature reports suggesting that school-age children no longer present difficulties in this area [4,31]. This is the first study that simultaneously investigates the ability to discriminate differences between frequencies and speech sounds (phonemes) in young children. This study used as many as 18 phonological oppositions in terms of differences as to the place and manner of articulation, voicing and nasalization, while other studies limited the analysis of differences to 3–4 contrasts [32]. The use of abstract linguistic material in this study—nonsense words devoid of lexical and semantic load—makes the obtained results universal in relation to other language systems, especially since categorical perception is an auditory function that develops in children of the same age, regardless of language systems in which they grow up. Additionally, this research is valuable due to the large size of the examined group (much larger than in previous studies on the subject of APD) and the unique opportunity to compare the development of phoneme discrimination in typically developing school-age children and children with auditory processing disorders. Table 8 below presents previous investigations in the field of phoneme discrimination in different language systems. The table shows the number of study participants, age groups, the number of phonological oppositions tested, and the mother tongue of participants of the conducted study.

**Figure 3:**
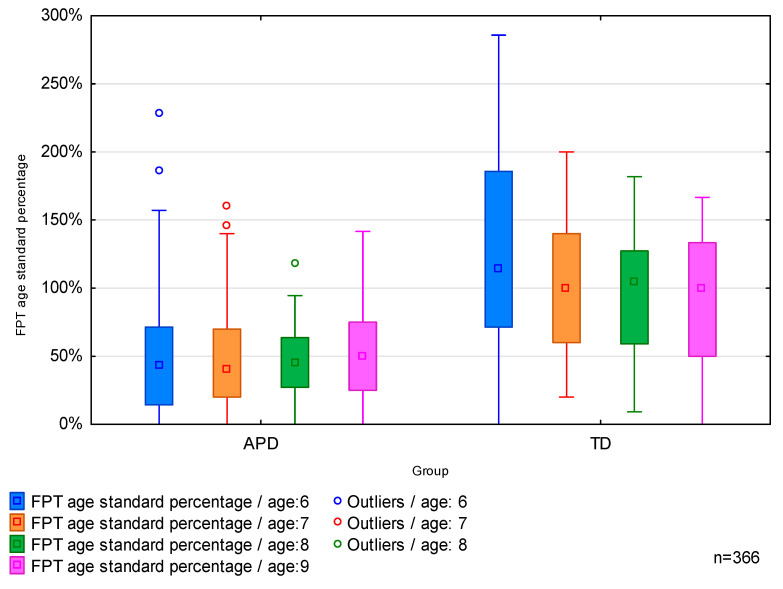
Results of the FPT test (age standard percentage), in 6, 7, 8, and 9-year-olds, in APD and TD groups.

This study showed that identified FPT deficits correspond with the occurrence of APD. The results were consistent across all APD age groups (Figure 3). While the FPT median results (calculated as a percentage of the age norm) of APD participants was 45%, it reached 100% for typically developing children. This means that in this test, children with APD achieved results more than twice as low as TD children. These differences are statistically significant in each of the studied age groups.

**Figure 4:**
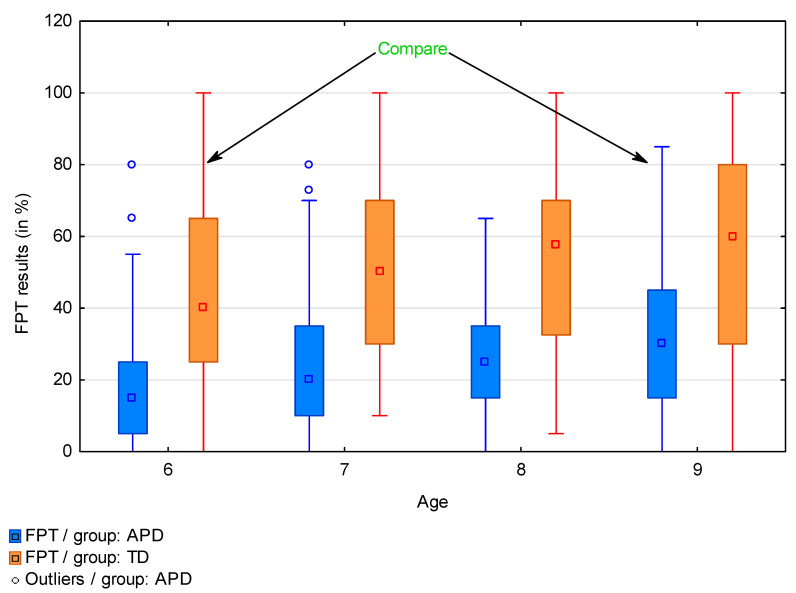
Comparison of the FPT results in APD and TD groups by age.

The results of the FPT, similarly to other auditory functions, improve with age in both groups. This is related to the physiological maturation of not only the central nervous system but the auditory pathway as well [21]. Despite this, 9-year-old APD participants achieved significantly worse test results than typically developing children at the age of 6. The median of the results of 6-year-olds in the TD group reached 40%, while the median of 9-year-olds from the APD group was only 30% (Figure 4, median values in Table 3 for comparison). These observations are consistent with the results of another study by the authors presenting deficits in the auditory functions of APD children and the dynamics of the development of auditory functions of typically developing children [24].

The results of the PDT showed that APD children have lower skills in the discrimination of phonemes than their peers without APD, and these differences are statistically significant. As with the results of the FPT, 9-year-olds with APD achieved lower results in the PDT than typically developing 6-year-olds. The median of the PDT results of 9-year-olds with APD was 22, while that of typically developing 6-year-olds was 23. The gradual improvement with age of the PDT results is observed in both research groups, similar to the FPT results.

The SD value of the mean results of the PDT decreases with age in the TD group. This indicates that the ability to discriminate phonemes is gradually equalizing among peers. However, in this group, this skill can still cause some difficulties even at the age of 9 (cf. Table 5). The SD value in the APD group does not decrease linearly, showing the imbalanced development of children from this group, which is apparently related to other difficulties APD children are struggling with and a possible impact of various therapeutic interventions implemented in this group. Therefore, age is not a dominating factor here.

Our results are consistent with the observations of other researchers conducting studies on different populations, various age groups and other languages [7,30,32]. The age at which the ability of phoneme discrimination fully matures, allowing the correct discrimination of phonological oppositions in the native language, has not yet been clearly determined. The research results indicate that the sensitivity to various phonological contrasts sharpens even as late as the teenage years.

In the study of phoneme discrimination by Attoni et al. [4] on a group of 46 younger children, including 24 typically developing participants and 22 with phonological disorders, aged from 5 to 7, the TD participants correctly identified all phonological oppositions in 30 pairs (organized into 40 presentations), including words with the same syllabic structure differing by only one phoneme (test with figures). While the participants with phonological disorders made mistakes, their results were not very poor, according to the authors’ comments. The mean of the results was 34, while in the TD group: 40. Attoni [4] did not differentiate the results by age group. Similarly, TD children from the age of 5, examined by Tamashige et al. [31], correctly differentiated phonemes in tests with words. Our study was based on nonsense words, and—while the TD participants did make mistakes in the PDT, the median of obtained results was always higher than those of APD participants. It may indicate that tests with words are too easy even for younger children and, therefore, not sensitive enough to detect discrete but clinically significant difficulties in phonemes discrimination.

The results of Edwards et al. [33] indicate that children with larger receptive vocabularies, regardless of age, achieve higher results in speech perception tests. Similarly, in Freitas et al. [15] study conducted on a group of 36 children aged from 5 to 8 years, a strong positive correlation was found, in the group of participants with developmental phonological disorders, between the level of phoneme discrimination and the language proficiency, in different linguistic subsystems. However, in the TD group, no such correlation was found. The TD participants easily obtained maximum results in the PDT. The authors suggest that such results may derive from the too-low difficulty of the PDT.

Neijenhuis et al. [22] examined a group of 105 TD children aged from 9 to 16. She analyzed the results obtained in both the FPT and PDT in two broad age groups: the first one—consisting of 75 participants aged from 9 to 12, and the second one—covering 30 examined, aged between 14 and 16. No significant age-related effects were found in the FPT or the categorical speech perception test. However, in the tests of categorical perception: discrimination and identification phonemes, only two phonological contrasts, stimuli from the [b-d] and [b-p] continuum, were used. The above authors state that the lack of age-related effects may result from early maturation of categorial perception, especially since in another study by Neijenhuis et al. [34] involving younger children (6 to 8 years old), the influence of age on the results of PDT was observed.

Thus, it seems reasonable to use abstract linguistic material for the assessment of the maturity of the sound of speech distinguishing ability of school-age children. Nonsense word tests, devoid of the influence of lexical and semantic factors, make the results of such tests independent of the language proficiency of a child [30,35]. Simultaneously, this approach eliminates the ceiling effect observed by researchers using tests with words and allows identifying children with even slight, although not obvious, difficulties in auditory perception. The results obtained in nonsense word tests would allow the development of dedicated therapeutic programs focused on specific deficits of particular patients. Our study shows that 6-year-olds usually had no difficulty with understanding the principle of the PDT using abstract linguistic material, in which the response procedure ‘same-different’ was used. Following simple instructions, supported by the presentation of three examples, they were able to obtain reliable test results in such tests.

An extensive study searching for a relationship between auditory functions and speech development was conducted by Kurkowska [36]. She examined three groups of children, aged from 7 to 9, distinguishing participants with delayed speech development, articulation disorders and TD participants. The test with words was used to examine phoneme discrimination skills. She found a statistically significant correlation between the results of the FPT and PDT only in the TD group (r = 0.42). In the remaining two groups, the correlations were much weaker compared to our results and were not statistically significant (r = 0.17 and r = 0.07, respectively). In Kurkowska’s [36] study, 9-year-old children with delayed speech development achieved significantly lower results in the FPT test than typically developing 7-year-olds. This pattern is comparable with our results obtained in the current and previous studies [24], so it allows for a preliminary comparison of the results. Kurkowska [36] found correlations between the results of the FPT and the general level of language proficiency. She noted the insufficient sensitivity of the PDT as a possible reason for the lack of correlation between the results of the FPT and PDT obtained by participants with delayed speech development and impaired articulation. Kurkowska [36] did not correlate the results of the PDT with age, perhaps due to the insufficient number of participants in particular age groups. She did not present the analysis of the PDT results in each age group, either.

Attoni et al. [4] found a relationship between the results obtained in tests evaluating auditory processing (she analyzed, among others, the results of dichotic tests) and the ability to discriminate phonemes. She recommended that the diagnostic process of speech and language disorders should include a phoneme discrimination test and an auditory processing assessment. The shortcoming of Attoni’s [4] study was the very small number of participants and, therefore, a lack of analysis by age. Similar recommendations were presented by Rouillon et al. [20], who compared the results of tests evaluating auditory functions and the level of phoneme discrimination in children with and without APD. Based on the results, she postulated the inclusion of the PDT into the APD diagnostic battery, especially if the patients do not show associated specific language disorders. The latter study was conducted on a group of 38 children aged 6 to 17 years, and the APD subgroup was not homogenous; children had comorbid conditions. Phoneme discrimination was assessed using only one phonological opposition: [də] and [tə].

The results of our study showed correlations between the level of phoneme discrimination and the results of the FPT in both groups. Although the correlations were assessed as fair, it should be emphasized that they were statistically significant, and their strength was very similar. We assume that the observed phenomenon is related to the independence of the results of the PDT from the language level of the participants—due to the use of abstract linguistic material. Thus, it makes the results universal for other language systems.

## 5. Limitations and Future Directions

The present study faced several limitations. The capacity of working memory, which is one of the factors affecting the results of the FPT, was not assessed in our study. Future research is needed to investigate the influence of mental abilities on FPT and PDT scores.

Additionally, it would be worth conducting an analogous study on a group of children with clearly diagnosed SLI and comparing such results with the ones of APD participants, e.g., assuming the results obtained in this study as a reference.

The complex statistical models widely used in neuropsychological research were not applied in this study. The authors are aware that more sophisticated tests can be used to analyze the data and plan to include analysis with linear mixed-effects models in future research [37].

## 6. Conclusions

Numerous articles concerning APD and SLI participants were focused on the analysis of frequency discrimination [11,38,39,40,41,42,43,44]. However, only a few studies have been focused on the analysis of the discrimination of phonemes [20]. Our research fills this gap. Although the difficulty in distinguishing phonemes is a well-known symptom of APD, there are no studies available in the literature that extensively illustrate this deficit. Our research conducted on a large group of participants confirmed the hypothesis that children with APD have more difficulties with phoneme discrimination than their typically developing peers. This study shows that in both the frequency and phoneme discrimination tests, 9-year-olds with APD did not reach the level of typically developing 6-year-olds. Additionally, we noticed:

1. The development of phoneme discrimination does not end with the acquisition of phonemes (around 6 years of age) but continues during school age;
2. The depth of the deficits observed through the results of the FPT test corresponds to the occurrence of auditory processing disorders. The participants with APD obtained results twice as low in the FPT test as their typically developing peers.

The obtained results indicate the need to include a phoneme discrimination test in the APD diagnostic procedure. Tests using abstract linguistic material (nonsense words) make the results independent of the language proficiency of the participants. Simultaneously, these tests are sensitive enough to capture the difficulties with phoneme discrimination that school-age children may experience and which may cause their educational failure. The inclusion of the PDT into the test battery for APD diagnostics will allow for the development of detailed therapeutic recommendations precisely adapted to the specific difficulties of children with APD.

## Figures and Tables

**Table 1:** Auditory processing disorder (APD) and typically developing (TD) groups: sex distribution by age.

	Group	APD			TD		
Age (Years)		Females	Males	Total	Females	Males	Total
6		20 (34%)	39 (66%)	59	9 (26%)	25 (74%)	34
7		17 (31%)	37 (69%)	54	15 (38%)	24 (62%)	39
8		14 (27%)	38 (73%)	52	7 (22%)	25 (78%)	32
9		18 (33%)	37 (67%)	55	20 (49%)	21 (51%)	41
Total		69 (31%)	151 (69%)	220	51 (35%)	95 (65%)	146

**Table 2:** Interpretation of correlation coefficient values.

Correlation Coefficient Value	Dependence Interpretation
below 0.3	poor
0.3–0.5	fair
0.6–0.8	moderately strong
min. 0.8	very strong

**Table 3:** Basic statistics describing the Frequency Pattern Test (FPT) results in the APD and TD groups by age.

FPT Results (%)											
		N		Mean (SD)		Median		Min		Max	
	Group	APD	TD	APD	TD	APD	TD	APD	TD	APD	TD
Age											
6		59	34	19.32 (19.87)	42.94 (27.94)	15	40	0	0	80	100
7		54	39	22.65 (18.49)	50.77 (26.55)	20	50	0	10	80	100
8		52	32	25.44 (15.39)	52.66 (25.34)	25	58	0	5	65	100
9		55	41	32.64 (20.48)	53.61 (28.71)	30	60	0	0	85	100
Total		220	146	24.91 (19.25)	50.16 (27.27)	20	50	0	0	85	100

**Table 4:** Basic statistics describing the results of FPT, presented as a percentage of age standard, in APD and TD groups, by age.

FPT Results (as a Percentage of Age Standard)											
		N		Mean (SD)		Median		Min		Max	
	Group	APD	TD	APD	TD	APD	TD	APD	TD	APD	TD
Age											
6		59	34	55 (57)	123 (80)	43	114	0	0	229	286
7		54	39	45 (37)	102 (53)	40	100	0	20	160	200
8		52	32	46 (28)	96 (46)	45	105	0	9	118	182
9		55	41	54 (34)	89 (48)	50	100	0	0	142	167
Total		220	146	50 (41)	102 (59)	45	100	0	0	229	286

**Table 5:** Basic statistics describing the results of the Phoneme Discrimination Test (PDT), in APD and TD groups, by age.

Phoneme Discrimination Test Results											
		N		Mean (SD)		Median		Min		Max	
	Group	APD	TD	APD	TD	APD	TD	APD	TD	APD	TD
Age											
6		59	34	18.86 (3.9)	22.24 (2.5)	20	23	7	16	24	25
7		54	39	20.59 (3.1)	22.54 (2.6)	21	23	12	14	25	25
8		52	32	21.46 (2.5)	23.16 (1.5)	22	24	15	19	25	25
9		55	41	21.22 (3.2)	23.80 (1.3)	22	24	11	20	25	25
Total		220	146	20.4 (3.37)	23 (2.1)	21	24	7	14	25	25

**Table 6:** Analysis of differences between age groups for the PDT results, in APD and TD groups, with the application of the post hoc tests (multiple comparisons tests of mean ranks for all samples).

Group	APD	TD
Significance level	*p* < 0.000	*p* = 0.025
Differences between age groups	6 and 8-year-olds (*p* = 0.000) 6 and 9-year-olds (*p* = 0.000)	6 and 9-year-olds (*p* = 0.029)

**Table 7:** Correlations between the results of the FPT and PDT for the TD and APD groups.

Spearman’s Correlation Coefficient		Pearson’s Correlation Coefficient	
TD	APD	TD	APD
r = 0.27	r = 0.23	r = 0.26 *p* = 0.002 r2 = 0.07	r = 0.24 *p* = 0.000 r2 = 0.09

**Table 8:** Studies on phoneme discrimination development (TD participants and participants with phonological disorders or APD).

Studies	Sample Size	Age of Participants	Number of Phonemes	Language
1. Attoni, T.M. et al., 2010 [4]	TOTAL: 46 control group: 24 (TD) study group: 22 (with phonological disorders)	5–7	30	Portuguese
2. Tamashige, E. et al., 2008 [31]	TOTAL: 211 (TD)	2;6–5;11	16	Japanese
3. Freitas, C.R. et al., 2015 [15]	TOTAL: 36 (including TD and phonological disorders)	5;3–7;11	30	Portuguese
4. Neijenhuis, K. et al., 2002 [22]	TOTAL: 105 (TD)	8;6–16;4	2	Dutch
5. Rouillon, I. et al., 2021 [20]	TOTAL: 38 APD: 17 no APD: 17 uncertain diagnosis: 4	6–17 (average and median: 9)	1	French
6. Guzek, A.; Iwanicka-Pronicka, K.	TOTAL: 366 APD: 220 TD: 146	6;0–8;11	18	Polish

## Data Availability

The data presented in this study are openly available in the Figshare.com repository at doi:10.6084/m9.figshare.22179614.

## Appendix A

The procedure of testing higher auditory functions, described in the previous article of the authors [24]

1. „ASPN-S test: a test assessing understanding of speech in noise. It involves presenting monosyllabic words in the presence of noise (the presented study used a multitalker babble). Monosyllabic words were presented binaurally with variable signal intensity, together with multitalker babble, which was presented with a constant intensity of 60 dB. The task of the listener was to repeat the heard word. The correct answer was considered to be a precise repetition of the word, while the incorrect—no repetition or inaccurate repetition (e.g., saying a different word). The first two words were given with a large difference between the intensity of the signal and the noise (14 dB), which allowed the test participant to answer with confidence. Each subsequent word was delivered 4 dB lower until the listener answered incorrectly. Following the incorrect response, the intensity of the ”word” stimulus was increased by 4 dB, and the stimulus was continued until the correct response was obtained. Subsequently, the intensity of the stimulus was decreased by 2 dB. These changes in intensity were repeated 5 times. The mean signal-to-noise ratio (SNR) threshold was determined based on the 4 lowest values at which the participant gave the correct answers [45].
2. DDT: a test of dichotic listening with distracted attention. It involves presenting 20 sequences of two different pairs of numbers (1–10) to the left and right ear at the same time. The participant’s task is to repeat all four numbers heard in a sequence; the order of the numbers is not relevant. The result is presented as a percentage of correctly recognized sequences for each ear, respectively. This test assesses the maturity of the auditory system in its central part and enables the identification of cerebral hemisphere dominance in terms of verbal stimuli;
3. FPT: a test checking the frequency pattern of sequences. It involves the determination of the sequence of the sounds heard. Twenty sequences of three-element tones of two frequencies (high tone: 1020 Hz, low tone: 880 Hz) were presented. The participant’s task was to recognize and name the heard sounds in the presented order, e.g., when the sounds of the high, low, and high pitch were presented, the child was expected to answer: high, low, high. The result of the test was the percentage of correctly recognized sequences. FPT can be used for assessing the ability to discriminate sound frequencies, level of auditory short-term memory and level of functioning of the right cerebral hemisphere.”

## Appendix B

**Table A1:** A list of nonsense words to assess the ability to discriminate oppositional phonemes of the Polish language (autors’ own work).

No.	Oppositive Phonemes Orthographic Polish Transcription	Oppositive Phonemes Slavistic Phonetic Alphabet (SPA)		Oppositive Phonemes International Phonetic Alphabet (IPA)		Phonological Opposition
1	Lasia–lacia	[laśa]–[laća]	[ś–ć]	[laɕa]–[laʨ̑a]	[ɕ–ʨ̑]	manner of articulation
2	zela–żela	[zela–žela]	[z–ž]	[zɛla]–[ʒɛla]	[z–ʒ]	place of articulation
3	abo–abo *	[abo–abo] *	–	[abɔ–abɔ] *	–	–
4	dzal–zal	[ʒal–zal]	[ʒ–z]	[ʣ̑al–zal]	[ʣ̑–z]	place of articulation
5	pecul–pesul	[pecul–pesul]	[c–s]	[pɛcul–pɛsul]	[c–s]	manner of articulation
6	ogat–ogat *	[ogat–ogat] *	–	[ɔgat–ɔgat] *	–	–
7	esak–eszak	[esak–ešak]	[s–š]	[ɛsak–ɛʃak]	[s–ʃ]	place of articulation
8	furi–wuri	[furi–vuri]	[f–v]	[furi–vuri]	[f–v]	voicing
9	taso–taso *	[taso–taso] *	–	[tasɔ–tasɔ] *	–	–
10	naś–niaś		[n–ń]		[n–ˈɲ]	place of articulation
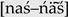
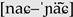
11	poj–boj	[poi̯–boi̯]	[p–b]	[pɔj–bɔj]	[p–b]	voicing
12	dzek–dzek *	[ʒek–ʒek] *	–	[ʣ̑ɛk–ʣ̑ɛk] *	–	–
13	kocz–koć	[koč–koć]	[č–ć]	[kɔʧ̑–kɔʨ̑]	[ʧ̑–ʨ̑]	place of articulation
14	sełe–zełe	[seu̯e–zeu̯e]	[s–z]	[sɛwɛ–zɛwɛ]	[s–z]	voicing
15	tech–tęch	[teχ–teŋχ]	[e–eŋ]	[tɛx–tɛŋx]	[ɛ–ɛŋ]	vowels / nasalization
16	simo–simo *	[śĩmo–śĩmo] *	–	[ɕĩmo–ɕĩmo] *	–	–
17	inej–imej	[ˈinei̯–ˈimei̯]	[n–m]	[ˈinɛj–ˈimɛj]	[n–m]	place of articulation
18	cape–cate	[cape–cate]	[p–t]	[ʦ̑apɛ–ʦ̑atɛ]	[p–t]	place of articulation
19	olit–olit *	[olʹit–olʹit] *	–	[oˈlʲit–oˈlʲit]	–	–
20	pofa–pąfa	[pofa–poŋfa]	[o–oŋ]	[pofa–pɔȵfa]	[o–ɔȵ]	vowels / nasalization
21	siepi–ziepi	[śepi–źepi]	[ś–ź]	[ɕĩɛpi –ˈʑĩɛpi]	[ɕĩ –ˈʑĩ]	voicing
22	tąsy–tąsy *	[toų̯sy–toų̯sy] *	–		–	–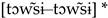
23	koczy–kodży	[kočy–koǯy]	[č–ǯ]	[kɔʧ̑ɨ–kɔʤ̑ɨ]	[ʧ̑–ʤ̑]	voicing
24	zurka–zulka	[zurka–zulka]	[r–l]	[zurka–zulka]	[r–l]	manner of articulation
25	praki–plaki	[praḱi–plaḱi]	[r–l]	[praci–placi]	[r–l]	manner of articulation

* No difference.

## Appendix C

**Table A2:** Tests for normality to verify the distribution of data. Test value (p) below 0.05 (marked in red in the table) indicates rejection of the null hypothesis (H0) assuming a normal distribution (autors’ own work).

Group APD: Kolmogorov–Smirnov Test and Lilliefors Test										
Age	Total		Age: 6		Age: 7		Age: 8		Age: 9	
Test	KS	LF	KS	LF	KS	LF	KS	LF	KS	LF
**FPT**	<0.01	<0.01	<0.10	<0.01	>0.20	<0.05	>0.20	>0.20	<0.20	<0.01
**FPT (%)**	<0.05	<0.01	<0.10	<0.01	>0.20	<0.05	>0.20	>0.20	<0.20	<0.01
**PDT**	<0.01	<0.01	<0.15	<0.01	<0.05	<0.01	<0.10	<0.01	<0.10	<0.01
**Group TD: Kolmogorov–Smirnov test and Lilliefors test**										
**Age**	**Total**		**Age: 6**		**Age: 7**		**Age: 8**		**Age: 9**	
**Test**	**KS**	**LF**	**KS**	**LF**	**KS**	**LF**	**KS**	**LF**	**KS**	**LF**
**FPT**	<0.20	<0.01	>0.20	<0.20	>0.20	>0.20	>0.20	>0.20	>0.20	<0.05
**FPT (%)**	>0.20	>0.20	>0.20	<0.20	>0.20	>0.20	>0.20	>0.20	>0.20	<0.05
**PDT**	<0.01	<0.01	<0.05	<0.01	<0.05	<0.01	<0.05	<0.01	<0.05	<0.01
**Group APD and TD: Shapiro-Wilk test**										
**Age**	**Total**		**Age: 6**		**Age: 7**		**Age: 8**		**Age: 9**	
**Test**	**Group APD**	**Group TD**	**Group APD**	**Group TD**	**Group APD**	**Group TD**	**Group APD**	**Group TD**	**Group APD**	**Group TD**
**FPT**	0.000	0.001	0.000	0.160	0.000	0.094	0.105	0.418	0.006	0.020
**FPT (%)**	0.000	0.004	0.000	0.160	0.000	0.094	0.105	0.418	0.006	0.020
**PDT**	0.000	0.000	0.000	0.009	0.002	0.000	0.001	0.003	0.000	0.000

**KS—**Kolmogorov–Smirnov test; **LF**—Lilliefors test; **FPT**—Frequency Pattern Test; **FPT (%)**—Frequency Pattern Test presented as a percentage of the age standard PDT—Phoneme Discrimination Test.

## Appendix D

**Table A3:** Reference values for the APD test results [45,46].

Age	DDT RE	DDT LE	ASPN-S	FPT
6	65%	45%	SNR 0 dB	35%
7	65%	50%	SNR −1 dB	50%
8	75%	60%	SNR −2 dB	55%
9	75%	60%	SNR −2 dB	60%

The interpretation of the results of tests:

− DDT RE, DDT LE, FPT: the value was considered to be normative when the result obtained was higher than the reference value for the age group,
− ASPN-S: the value was considered to be normative when the result obtained was lower than the reference value for the age group.
